# The Interplay between Uremic Toxins and Albumin, Membrane Transporters and Drug Interaction

**DOI:** 10.3390/toxins14030177

**Published:** 2022-02-26

**Authors:** Regiane Stafim da Cunha, Carolina Amaral Bueno Azevedo, Carlos Alexandre Falconi, Fernanda Fogaça Ruiz, Sophie Liabeuf, Marcela Sorelli Carneiro-Ramos, Andréa Emilia Marques Stinghen

**Affiliations:** 1 Experimental Nephrology Laboratory, Basic Pathology Department, Universidade Federal do Paraná, Curitiba 81531-980, Brazil; regidacunha@ufpr.br (R.S.d.C.); carolina.amaral1@ufpr.br (C.A.B.A.); 2 Laboratory of Cardiovascular Immunology, Center of Natural and Human Sciences (CCNH), Federal University of ABC, Santo André 09210-580, Brazil; falconi.familia@gmail.com (C.A.F.); fernandafogaca1@gmail.com (F.F.R.); marcela.ramos@ufabc.edu.br (M.S.C.-R.); 3 Department of Pharmacology, Amiens University Medical Center, 80054 Amiens, France; liabeuf.sophie@chu-amiens.fr

**Keywords:** uremic toxins, albumin, cell transporters

## Abstract

Uremic toxins are a heterogeneous group of molecules that accumulate in the body due to the progression of chronic kidney disease (CKD). These toxins are associated with kidney dysfunction and the development of comorbidities in patients with CKD, being only partially eliminated by dialysis therapies. Importantly, drugs used in clinical treatments may affect the levels of uremic toxins, their tissue disposition, and even their elimination through the interaction of both with proteins such as albumin and cell membrane transporters. In this context, protein-bound uremic toxins (PBUTs) are highlighted for their high affinity for albumin, the most abundant serum protein with multiple binding sites and an ability to interact with drugs. Membrane transporters mediate the cellular influx and efflux of various uremic toxins, which may also compete with drugs as substrates, and both may alter transporter activity or expression. Therefore, this review explores the interaction mechanisms between uremic toxins and albumin, as well as membrane transporters, considering their potential relationship with drugs used in clinical practice.

## 1. Introduction

Kidney diseases were the 10th leading cause of death worldwide in 2019, according to the World Health Organization (WHO). Their mortality rate has increased approximately 37.4% in 19 years. One of the main consequences of the loss of renal function is an accumulation of uremic toxins in the body, affecting the various tissues and organs, including the cardiovascular system [1]. The biological effects promoted by uremic toxins depend on the relationship between production, degradation, and excretion, in addition to cytoplasmic distribution and the presence of inhibiting or promoting agents of the toxin’s action [2].

The European Uremic Toxin Work Group (EUTox) reports that uremic toxins can be classified into three groups due to their physicochemical characteristics and their behavior during dialysis [1]: (I) Small-water soluble compounds (molecular weight <500 Da), such as creatinine and urea; (II) Medium compounds (peptides with molecular weight >500 Da), such as cystatin-C and β_2_-microglobulin, which can only be removed by large pore size dialysis membranes; and (III) Protein-bound uremic toxins (PBUTs), such as indoles and phenols, which come from dietary amino acid metabolism and are poorly filtered by the dialytic membrane.

Most of the small-water soluble compounds are well known, and some can be used in the diagnosis of kidney diseases, such as serum creatinine and blood urea nitrogen (BUN) which are classic biomarkers in the progress of chronic kidney disease (CKD). Trimethylamine-*N*-oxide (TMAO) is in this same group, a uremic toxin associated with an increased risk of developing cardiovascular diseases (CVD), including cardiac dysfunction and atherosclerosis [3,4]. Another small-water soluble compound is inorganic phosphorus (Pi), which may not be considered as uremic toxin for some authors, but has a clear role in CVD progression. Hyperphosphatemia has been associated with accelerating the progress of renal dysfunction and is also correlated with a higher mortality rate from CVD and peripheral and visceral vascular calcification [2].

It is important to mention fibroblast growth factor 23 (FGF-23), β_2_-microglobulin, parathyroid hormone (PTH), and pro-inflammatory molecules such as interleukin-6 (IL-6) among the medium compounds [5,6]. High levels of these toxins contribute to progressive renal structural damage; however, they are fundamental to mineral homeostasis maintenance in a healthy organism.

PBUTs stand out for their high affinity for proteins, particularly serum albumin, making their removal by dialysis therapies difficult. Tubular secretion plays a key role in the renal elimination of these uremic toxins, with the residual renal function being an important factor in uremic levels in patients with advanced CKD [7]. Regarding PBUTs, it is important to highlight that there are few studies that have addressed these molecules, and they demand attention from the scientific community due to their behavior during dialysis, for example [5,8,9]. Given the relevant role of uremic toxins in CKD, in the next topic, we address the main PBUTs, followed by how the interaction with albumin occurs.

## 2. Protein-Bound Uremic Toxins (PBUTs)

### 2.1. Indoxyl Sulfate (IS)

Indoxyl sulfate (IS) constitutes one PBUT and is a product of the bacterial metabolism of dietary tryptophan by bacteria in the gut and converted to indole, which crosses the intestinal barrier and reaches the liver where it is converted to indoxyl, and later sulfated to IS ions, in the way it is found in the bloodstream and tissue of patients with compromised renal function [10,11,12]. About 90% of it in blood plasma is primarily bound to serum proteins such as albumin, and this binding causes its excretion to primarily occur by proximal tubular secretion and then by glomerular filtration [13].

Patients with CKD have a total IS concentration surpassing 500 μM compared to 0.1–2.39 μM in patients with healthy kidney functions [14]. As previously mentioned, the dialytic membrane pores do not effectively remove IS since 90% of it is bound to serum albumin, making the complex too large to be filtered. This retention is associated with diverse harmful effects in other organs, such as alterations to thyroid function, endothelial dysfunction, smooth muscle cell proliferation, and atherosclerosis [15,16]. IS is related to many harmful effects to the organism, with a hypertrophic effect in cardiomyocytes through the activation of the mitogen-activated protein kinase (MAPK) and nuclear factor-κB (NF-κB) pathways among them, in turn indicating that this toxin has a crucial role in developing cardiac hypertrophy under uremic conditions [17,18]. Another effect of IS is the activation of proinflammatory macrophages which generate an immune dysfunction. This activation is mediated by the uptake through transporters, including OATP2B1, which is an important mediator of inflammatory process signaling [19].

### 2.2. p-Cresyl Sulfate (PCS)

*p*-Cresyl Sulfate (PCS) is another PBUT generated from the metabolism of tyrosine and phenylalanine, two aromatic amino acids which are metabolized by bacteria from intestines. This molecule has a low molecular weight (108 Da), as with IS [2,5]. *p*-Cresol suffers sulfation and glucuronidation in the mucosa in the distal part of the colon of the large intestine and liver in the degradation process, generating PCS and *p*-cresyl-glucoronate [20]. As *p*-cresol is promptly metabolized, uremic patients show normal levels of these compounds similar to healthy people. Thus, PCS is the conjugated form of *p*-cresol with evident retention in the bloodstream of patients with CKD [21]. Serum concentration rates range from 2.8 ± 1.7 mg/L (14.9 ± 9.0 µM) 7 and 6.6 ± 3.7 mg/L (35.1 ± 19.7 µM) in patients without serious renal impairment. These concentrations in patients with end-stage CKD can range from 21.8 ± 12.4 mg/L (115.8 ± 65.9 µM) to 106.9 ± 44.6 mg/L (568.0 ± 237.0 µM), both quantified by UPLC in serum and LC-MS-MS in plasma, respectively [20,21].

Several studies point out the damage caused by PCS accumulation, such as smooth muscle cell lesions, endothelial dysfunction, coagulation disturbances, leukocyte activation, cardiac fibrosis, and metabolic disorders, including insulin resistance [21,22]. Other works have shown a deleterious effect of PCS in specific renal and cardiac cells, contributing to decrease glutathione levels promoting redox unbalance [23]. Consequently, it is possible to observe cardiac dysfunction, facilitating cardiomyocyte apoptosis and mitochondrial hyperfusion [8,24]. All effects demonstrate that this compound is linked to cardiovascular damage and contributes to the increase in mortality and cardiovascular events in CKD [2,25].

### 2.3. Indole-3-Acetic Acid (IAA)

In addition to IS, indole-3-acetic acid (IAA) is a PBUT derived from the gut metabolism of dietary tryptophan with a molecular weight of 264.27 Da [26,27]. Tryptophan-derived uremic toxins are agonists of the aryl hydrocarbon receptor (AhR) complex, and their accumulation in patients with CKD may activate the AhR [28], which leads to pro-oxidant, pro-inflammatory, pro-coagulant, and pro-apoptotic effects. IAA can also induce cyclooxygenase-2, worsening the inflammatory state and increasing oxidative stress [29].

Beyond the classic and canonical actions of AhR activation, the non-canonical AhR signaling after IS or IAA stimulation is responsible for blocking the cell cycle and suppressing the S-phase genes. Some studies have shown the potential carcinogenesis control combined with an increase in inflammatory cytokine expression through NF-kB. Moreover, the activation of AhR can also promote proteolysis of the endoplasmic reticulum (ER), assembling the ubiquitin ligase complex [30].

IAA has been found to stimulate glomerular sclerosis and interstitial fibrosis, accelerating renal damage and the progression of CKD [31]. In a study with transplanted and non-transplanted patients with CKD, Liabeuf et al. (2020) demonstrated that free and total IAA gradually increased with CKD progression and that IAA levels were elevated at the transplant time but substantially decreased one month after transplantation [26]. Moreover, the free IAA level predicted overall mortality and cardiovascular events in the non-transplanted CKD cohort [26].

### 2.4. 3-Carboxy-4-methyl-5-propyl-2-furanpropionate (CMPF)

3-Carboxy-4-methyl-5-propyl-2-furanpropionate (CMPF) is one of the major metabolites of furan fatty acids and shows incredible protein-binding affinity reaching almost 100% binding with site I of albumin; thus, it is not removed by conventional dialysis therapies [32,33]. CMPF plasma levels can remain high even 90 days after a successful kidney transplant [34]. The source of elevated levels of circulating CMPF is still unknown, and the consequences of its accumulation are still unclear [35,36]. However, it was demonstrated that CMPF is elevated in diabetes and acts directly in β cells, dysregulating key transcription factors, eventually leading to reduced insulin biosynthesis, and inducing oxidative stress [37]. CMPF in CKD directly interacts with oxygen radicans and can enhance the production of reactive oxygen species (ROS) in HK-2 cells and consequently induce cell damage [38]. Given its role in oxidative stress, CMPF is often associated with uremic toxins with cardiovascular relevance [39,40]. However, a study conducted by Luce et al. (2018) with patients in hemodialysis showed that elevated serum CMPF levels were not associated with mortality or cardiovascular mortality in that cohort but were positively correlated with nutritional parameters and lean mass and is significantly elevated in patients without protein-energy wasting [41].

## 3. Interaction between Uremic Toxins and Albumin

Human serum albumin (HSA) is the most abundant protein in human plasma, representing 50–60% of the total plasma proteins, the main protein responsible for maintaining the colloid osmotic pressure of the blood. HSA is a 66 kDa monomer that predominantly adopts a heart-shaped tertiary structure, containing three homologous helical domains (I–III) divided into A and B subdomains. HSA displays incredible binding capacity, serving as a carrier for many endogenous and exogenous molecules such as fatty acids, hemin, thyroxin, bilirubin, and a wide variety of drugs such as warfarin, diazepam, and ibuprofen, which usually bind to one of the two primary sites [42,43,44]. HSA binding improves the plasma solubility of these drugs but also reduces their free active concentration [45]. HSA can bind to peptides and proteins under physiological conditions, which impacts proteomic and biomarker studies, as the presence of binding and unbinding forms of these proteins can affect their detection and clearance [46]. Lastly, HSA is known to wield antioxidant properties in plasma, being the major source of reduced sulfhydryl groups, which act as scavengers of reactive oxygen species (ROS) and reactive nitrogen species (RNS) and can limit the production of these reactive species by binding free Cu^2+^, an ion that is known to be important in quickening the production of free radicals [47]. In addition, increased oxidative stress can induce oxidative modifications of HSA, including glycation, disulfide bond formation, and carbonylation, which can alter its binding properties, thus increasing or decreasing ligand affinity [42,48,49,50].

Oxidative stress has a key role in CKD development and is already present in its early stages [51]. Enhanced oxidative stress substantially contributes to CVD complications and further impairment of renal function with CKD progression. The HSA molecule appears to be altered in patients undergoing hemodialysis, which results in impairment of its physiological activities [52] and presents low antioxidant activity, capable of inducing an oxidative burst of neutrophils [53,54]. Moreover, the oxidized HSA levels are significantly increased with the decrease of renal function [55].

Various CKD-associated metabolic disturbances may alter drug distribution and trigger a CKD stage- decrease in albumin drug binding. The small water-soluble molecule urea can lead to post-translational carbamylation of proteins and thus changes in the latter’s structure and function [56,57]. Indeed, plasma from CKD patients presents varying degrees of plasma protein carbamylation correlating with the values of free plasma salicylate [58].

**Figure 1:**
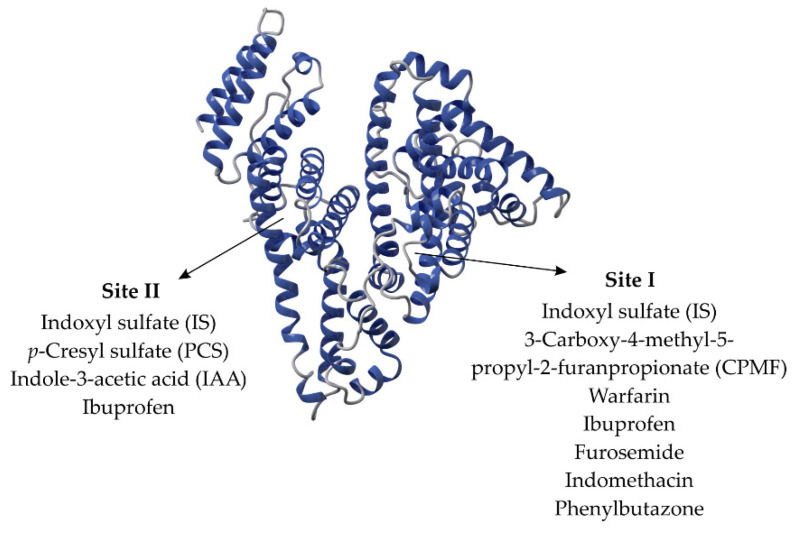
Albumin binding sites with PBUTs and drugs.

One of the main roles of HSA in the context of CKD is its capacity to bind with uremic toxins, especially PCS and IS. Recent studies report that IS is present in both I and II binding sites, with a preference for site II [43]. In contrast, PCS is present within site II [59,60] and possibly an unknown binding site, according to results achieved by Li et al. (2022) [43]. Figure 1 shows PBUTs and drugs that bind to sites I and II of albumin. Once in the bloodstream, about 85–95% of both IS and PCS bind to HSA. Albumin-toxin binding can be altered by the concentration of available albumin in plasma or by albumin updating due to factors such as heat [61].

Hirata et al. recently investigated the relationship between the binding of aripiprazole, an antipsychotic agent, and the concentration of uremic toxins. The binding of aripiprazole in the cases of renal failure was reduced significantly, compared with the same values for healthy adults. An association was found between the ARP binding rate and the concentration of toxins, including IS and PCS [62].

In a cohort of 403 kidney transplant recipients, André et al. reported that the blood tacrolimus concentration was significantly associated with plasma IS, PCS, and urea levels. The authors’ hypothesis was that IS and PCS bind to albumin with a high affinity and might directly compete for tacrolimus binding sites on the protein, whereas urea might carbamylate albumin and thus modify tacrolimus binding. The blood concentration of cyclosporine (which mainly binds to lipoprotein rather than albumin) was not associated with plasma IS, PCS, and urea levels [63].

The binding of these toxins to HSA is problematic because they form a complex which is too large to be removed by conventional dialysis treatment, leading to an accumulation of these toxins and (as mentioned above) diverse harmful effects to patients with CKD. Therefore, many treatment options have been described and studied in the last few years.

Meyers et al. (2004) described a mathematical model of the behavior of PBUTs during hemodialysis, relating their clearance directly with the free fraction. Thus, increasing the free fraction of PBUTs by disrupting their binding with albumin is one possible way to improve the removal of PBUTs [64]. Böhringer et al. (2015) modified hemodiafiltration (HDF) with the perfusion of hypertonic NaCl solution to increase the ionic strength (HDF-IPIS), and their results showed that some PBUTs were removed more efficiently, however only the free fraction of IS was significantly increased by this method [65]. Yamamoto et al. (2019) examined the efficacy of activated carbon in adsorbing circulating PBUTs through direct hemoperfusion (DHP) In vitro, with their results showing that activated carbon can drastically adsorb IS, PCS, and IAA from the blood of hemodialysis patients [66]. However, the study did not assess the safety and biocompatibility of activated carbon for clinical use, and therefore more studies are needed to check its safety and efficacy.

Shi et al. (2019) studied the effects of ionic strength, pH, and chemical displacers on the percentage protein binding (PB) of PBUTs, showing that the PB% decreased with increasing ionic strength, but only a few changes occurred with the increased pH (6.0 to 8.5) [67]. Regarding chemical displacers, they studied ibuprofen, warfarin, phenylbutazone, indomethacin, furosemide, oleic acid, linoleic acid, and docosahexaenoic acid (DHA), which are known to be typical displacers of HSA sites I or II. The results showed that PCS, IS, and IAA were easily dissociated from albumin by these chemicals. The PB% for CMPF, PCS, IS, and IAA was significantly decreased in the presence of free fatty acids, oleic acid, and linoleic acid. In addition, Tao et al. (2016) showed in vitro that a concentration of 1 mM of ibuprofen could increase the free fraction of IS and PCS by a threefold ratio in uremic plasma [68]. Madero et al. (2019) demonstrated that 800 mg of ibuprofen infused in the arterial bloodline between minutes 21 and 40 of conventional 4 h high-flux hemodialysis results in a 3-fold increase of the dialytic removal of IS and PCS, leading to a reduction of their serum levels, and which disappear after stopping the ibuprofen infusion [69]. There was no difference in the clearance levels of non-protein-bound toxins, such as urea and creatinine. Therefore, displacement of PBUTs is a very attractive method. However, not all PBUTs will be affected by the same displacer, leading to the concerns gathered by Van Biesen and Elliot (2019), such as the need for efficient and rapid clearance of the substances to be used as displacers. They also need to be inert because a combination of displacers is most likely to be necessary to displace a higher number of PBUTs [70].

Another method has recently revealed potential in removing PBTUs, namely liposomes. Shi et al. (2019) reported that the addition of liposomes to the dialysate significantly enhanced PBUTs removal without influencing the removal of small, water-soluble solutes [71]. In 2020, Shen et al. (2020) constructed linoleic acid-modified liposomes (LA-liposomes) as an indirect adsorbent in the dialysate [72]. The LA-liposomes showed good binding properties to the PBUTs, bilirubin, and bile acids. Additionally, the albumin binding of PBUTs was significantly inhibited by the addition of linoleic acid, enhancing the removal of PBUTs and showing the potential of combining indirect adsorbent (i.e., LA-liposomes) and a competitive displacer (i.e., LA) for removing protein-bound uremic toxins [72].

## 4. Cell Membrane Transporters of Uremic Toxins

**Figure 2:**
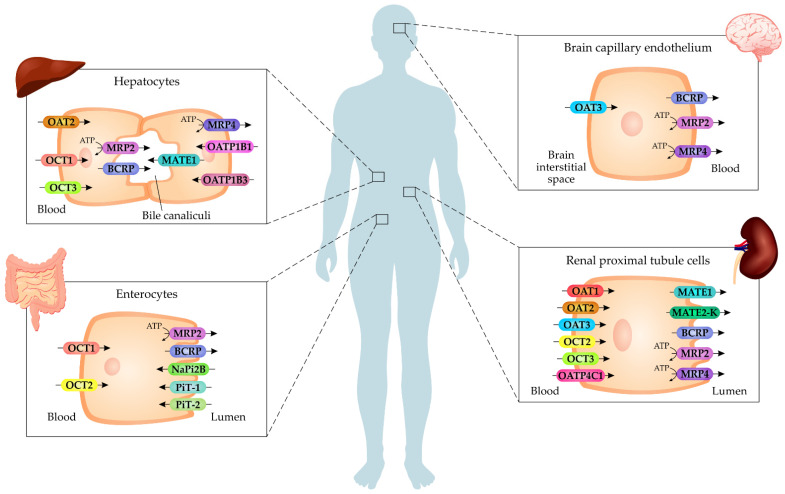
Cell membrane transporters that mediated transport of uremic toxins. Several transporters contribute to the cellular influx and efflux of uremic toxins across membranes, such as organic anion transporters (OATs), organic cation transporters (OCTs), organic anion-transporting polypeptides (OATPs), type-III sodium-dependent phosphate transporters (PiTs), multidrug and toxin extrusion (MATEs), the breast cancer resistance protein (BCRP), and multidrug resistance-associated proteins (MRPs). These transporters can be found in renal cells, hepatocytes, enterocytes, and endothelium.

Uremic toxins interact with membrane transporters, proteins which mediate the influx or efflux of these compounds into the cell. These toxins can activate signaling pathways upon entering the cell and modulate the cellular response under uremic conditions, contributing to the pathological process of CKD. The transport of uremic toxins across the cell membrane has been associated with representatives of the solute carrier (SLC) transporter and ATP-binding cassette (ABC) transporter superfamilies, which are known to transport a variety of endogenous and xenobiotic compounds and are also implicated in drug therapy. Importantly, membrane transporters are essential for the renal elimination of these compounds via tubular secretion. Therefore, it is suggested that the expression of these transporters may be related to the toxicity of uremic solutes that accumulate in the body with the CKD progression. A summary of cell membrane transporters that are involved in transporting or interacting with uremic toxins is presented in Figure 2 and Table 1.

### 4.1. Organic Anions Transporters (OATs)

Organic anion transporters (OATs) are polyspecific membrane transporters that perform the cellular influx of a wide variety of substrates, mainly organic anions, although some cations have also been identified as substrates [73,74]. Several uremic toxins, as well as many drugs, are organic anions that can compete for these transporters [75,76]. OATs belong to the SLC superfamily, specifically the SLC22A family. So far, the isoforms of OAT1-7, OAT10, and URAT1 have been identified in human tissues [77,78]. Regarding their structure, OATs have between 540 and 650 amino acids that are organized in 12 transmembrane α-helices domains with both NH_2_ and COOH terminations in the cytoplasm and multiple glycosylations in the extracellular loop [78].

OAT1 and OAT3, encoded by the *SLC22A6* and *SLC22A8* genes, respectively, are the most studied transporters of this family. Both proteins have an antiport-type transport mechanism in which substrates from the extracellular environment are captured in exchange for intracellular dicarboxylates [79,80]. They are found in the kidneys, mainly in the basolateral membrane of renal proximal tubule cells, where they participate in the tubular secretion of various compounds, including uremic toxins [77,80,81,82]. Furthermore, animal studies have shown that CKD may cause changes in the expression of OATs. These studies reported a decrease or no change in the expression of Oat1 and Oat3 in the kidneys of nephrectomized animals [83,84,85,86]. In contrast, nephrectomized rats treated orally with IS for ten weeks showed an increase in Oat1 levels in the renal tubules [87].

Metabolomic analyses have shown increased plasma levels of PCS, IS, and kynurenine uremic toxins in Oat1 knockout mice [88,89]. In addition to these same toxins, the Oat3 knockout mice also showed increased plasma CMPF and TMAO levels [89]. These data demonstrate that OAT1 and OAT3 are important for cellular uptake of several uremic toxins and are strongly related to their renal clearance. Some of these molecules have to be modified by phase 2 enzymes in the liver, demonstrating that OATs are key players in the gut-liver-kidney axis and suggesting an important role for OATs in proximal tubule metabolism [88,90,91]. Although OAT1 and OAT3 have overlapping substrates, literature data indicate that each one may have a greater or lesser contribution in the capture of specific substrates. PCS and IS seem to mainly be excreted by OAT3, while hippurate and IAA by OAT1 [92,93]. The basolateral uptake of PCS is inhibited by IS since its Km value is larger than the other, which may explain the high level of plasma concentration of this uremic toxin due to the competition in the urinary excretion in patients with CKD [93,94].

Several drugs also interact with OAT1 and OAT3, such as uricosuric agents, antivirals, β-lactam antibiotics, and non-steroidal anti-inflammatory drugs [95,96,97,98]. These drugs inhibit OATs in a competitive manner, such as probenecid and benzylpenicillin, or a non-competitive manner, as in the case of telmisartan, which alters the conformation of the transporter and impairs its activity [99]. The inhibition of OATs consequently affects the cellular uptake of uremic toxins in the kidneys and other tissues which express the OATs. Studies have shown that probenecid, a well-known OATs inhibitor, significantly reduces the uptake of PCS by kidney cells [93,100]. Similarly, Favretto et al. (2017) demonstrated that probenecid decreases both PCS and IS uptake by endothelial cells [101]. In vitro studies also indicate that inhibiting the entry of uremic toxins attenuates their biological effects on various cell types. Blocking OATs with probenecid re-established the inductive effect of uremic toxins on the expression of proinflammatory molecules by endothelial cells, such as monocyte chemoattractant protein-1 (MCP-1) and E-selectin [101,102,103]. Probenecid also inhibited the osteogenic differentiation of vascular smooth muscle cells (VSMCs) exposed to PCS [102]. IS-induced damage to osteoclasts and osteoblasts, such as apoptosis and dysfunction, was attenuated with probenecid [104,105]. IS promotes oxidative stress, cytokine expression, and atrophy in skeletal muscle cells, but these effects are reversed with probenecid [106]. All these data support the involvement of OATs in the entry of uremic toxins, such as PCS and IS, into the cell.

More recently, studies using animal models have investigated the use of drugs that inhibit OATs and their effect on uremic toxins levels, especially in the context of kidney disease. Luo et al. (2020) demonstrated that probenecid and ciprofloxacin reduced IS renal clearance by 89% and 71%, respectively, in rats treated with this uremic toxin [107]. Ciprofloxacin is an antibiotic that is related to the inhibition of OAT3-mediated transport [107,108]. In another study with an animal model, Li et al. (2021) observed that furosemide inhibited renal clearance of TMAO, increasing its plasma and kidney levels [109]. Studies have shown that furosemide, a diuretic drug, interacts with OATs and cell efflux transporters such as BCRP and MRP2 [89,109,110].

The accumulation of uremic toxins with the use of OATs inhibitors is also observed in humans. Granados et al. (2021) verified that a cohort of 20 human subjects treated with probenecid had elevated levels of tryptophan-derived metabolites, including IS and kynurenine [111]. In a cohort of kidney transplant patients, André et al. (2022) demonstrated that patients with a prescription of at least one drug which inhibits OAT1/OAT3 (*n* = 311) had higher plasma levels of IS, PCS, and IAA, but no difference in TMAO levels compared to patients without a prescription of OAT inhibitors (*n* = 92) [112]. The authors also showed a significant accumulation of PCS in patients with OAT-inhibiting drug prescriptions than in those without, even adjusting parameters for age, renal function, transplant time, and plasma albumin levels by multivariate analysis [112]. Together, these data indicate that drug pharmacokinetics may affect the transport of uremic toxins involving OATs, which highlights the need to understand the interaction of transporters with these drugs and the uremic toxins, especially in CKD.

OAT2 is another isoform of this protein family related to the uptake of uremic toxin creatinine. Renal clearance of creatinine is performed by glomerular filtration as well as tubular secretion, in which OAT2 participates together with other transporters [113,114]. OAT2 is encoded by the *SLC22A7* gene and is expressed in the kidneys and liver [77]. However, the transport of uremic toxins by other OATs is still unclear.

### 4.2. Organic Cation Transporters (OCTs)

Organic cation transporters (OCTs) are polyspecific transporters that mainly have cationic compounds as substrate, but they can transport other molecules depending on their properties [115,116]. Like OATs, OCTs also belong to the SLC22A family. The group presents the OCT1-3 isoforms, which are characterized by having from 540 to 560 amino acids in 12 transmembrane domains and several glycosylation and phosphorylation sites. The transport mechanism of OCT substrates is based on diffusion facilitated by electrogenic uniporters or as cation exchangers [115,117].

OCT2 is encoded by the *SLC22A2* gene and is related to the transport of uremic toxins, especially those from the group of guanidine compounds [116,118,119,120]. This transporter is found in the basolateral membrane of renal tubule cells, participating in the renal clearance of several endogenous and exogenous compounds [82,121]. Likewise, OCTs can interact with multiple drugs. For example, metformin is a drug widely used in diabetes treatment and a known substrate of OCT2 [122]. Cheung et al. (2017) investigated the interaction of OCT2 with 72 uremic solutes and identified that creatinine, dimethylamine, malondialdehyde, trimethylamine, homocysteine, indoxyl-β-d-glucuronide, and glutathione disulfide inhibited [^14^C]-labeled metformin uptake by OCT2, suggesting some type of interaction between these compounds and the transporter [123].

In vitro studies have also shown that creatinine, methylguanidine, guanidine, putrescine, and TMAO uremic toxins are substrates of OCT2 [110,116,118,124,125]. An approximately two-fold increase in plasma TMAO concentrations was observed in mice with double knockout of Oct1 and Oct2 compared to the control group [110,126]. Furthermore, the ratio between TMAO levels in kidneys and plasma were lower in mice with the Oct1/Oct2 knockout compared to the control group, but no difference was found in the ratio of TMAO levels in liver and plasma [110]. As Oct1 is expressed in the liver and Oct2 in the kidneys, these data indicate that Oct2 has an important role in the renal elimination of TMAO [110].

The interaction between drugs and OCT2 may influence the transport of uremic toxins. Studies have shown that serum creatinine levels may be increased with the use of drugs that compete for OCT2, impairing the tubular secretion of this uremic toxin [117,127]. Ciarimboli et al. (2012) reported that cancer patients undergoing treatment with cisplatin, also an OCT2 substrate, had elevated serum creatinine levels [128]. It was recently shown that the drug vandetanib inhibited cellular uptake of creatinine by OCT2 using mechanisms that are still unclear [129].

OCT2 expression in the kidneys is reduced in CKD. Studies with nephrectomized rats have shown a decrease in Oct2 levels in the kidneys [85,130]. However, Ji et al. (2002) demonstrated that Oct2 levels were restored in nephrectomized rats with the administration of testosterone, which regulates its expression [85]. Recently, Han et al. (2022) reported reduced levels of OCT2 in renal biopsies of patients with CKD [130]. In addition, polymorphisms in OCT2 gene encoding and changes in its expression may also influence the renal clearance via this transporter. Genomic studies have identified several single nucleotide polymorphisms (SNP) in intergenic or coding regions that are associated with OCT2 activity [128,131,132,133].

The transport capacity of uremic toxins related to other OCT isoforms is still unclear. OCT1 is encoded by the *SLC22A1* gene, is mainly found in the basolateral membrane of hepatocytes and may be involved in the uptake of guanidinovaleric acid [77,118]. On the other hand, OCT3 is encoded by the *SLC22A3* gene and widely distributed by tissues, including skeletal muscle, placenta, kidney, and epithelial cells of choroid plexus [77]. Although OCT3 is also found in the kidneys, its levels are lower than OCT2 [77,121]. However, OCT3 in the choroid plexus is related to the cellular uptake of creatinine and at least partly contributes to the removal of this uremic toxin from the cerebrospinal fluid [134].

### 4.3. Organic Anion-Transporting Polypeptides (OATPs)

Organic anion-transporting polypeptides (OATPs) belong to the organic solute carrier (SLCO) gene family, which is within the SLC superfamily. This group is characterized by mainly transporting amphipathic organic anions, including compounds larger than 300 Da, but they may also have neutral and positively charged molecules as substrates [79]. The structure of OATPs has 650 to 700 amino acids distributed in 12 transmembrane domains with post-translational modification sites and NH_2_ and COOH terminations in the cytoplasmic space [135]. Eleven isoforms of OATPs have been identified in human tissues with a wide variety of substrates, including endogenous compounds, toxins, and drugs [136].

OATP4C1 is encoded by the *SLCO4C1* gene and is highlighted by interacting with some uremic toxins and having its expression altered in CKD. This transporter is mainly found in the kidneys, in which it participates in excreting several molecules [77]. OATP4C1 mediates the cellular uptake of asymmetric dimethylarginine (ADMA), a uremic toxin known to contribute to endothelial dysfunction and CVD development in patients with CKD [137,138,139,140,141]. In vitro, Toyohara et al. (2009) observed that mice with transgenic-induced OATP4C1 expression in the kidneys showed a decrease in plasma levels of ADMA, suggesting that this transporter is involved in ADMA uptake [142]. Interestingly, nephrectomized rats treated with pravastatin, a drug from the statin group, had an increase in both OATP4C1 levels in the kidney and ADMA renal clearance [142]. In contrast, Akiyama et al. (2013) demonstrated that IS downregulated OATP4C1 expression in renal cells [143]. A reduction in OATP4C1 levels in the kidneys of rats treated with IS for 4 weeks compared to the control group was observed in vitro. This same study also demonstrated that rats treated with IS had an increase in serum guanidinosuccinate levels, an OATP4C1 substrate, but not of creatinine or ADMA [143]. Similarly, a decrease in OATP4C1 expression was observed in the kidneys of nephrectomized animals [144]. These data suggest that modulation of OATP4C1 expression by drugs may not only influence the transport of uremic toxins, but uremic toxins can also modulate the transporter expression.

Uremic toxins can also affect the expression and activity of OATPs present in the liver and, therefore, influence the metabolism of other compounds, including drugs. This is the case of OATP1B1 and OATP1B3, encoded by the *SLCO1B1* and *SLCO1B3* genes, respectively. Both transporters had their expression reduced in hepatocytes exposed to uremic plasma from patients with CKD [145]. The transporters also had their activity inhibited in the presence of the uremic toxin mix that included IS, indole acetate, hippuric acid, and CMPF [145]. The downregulation and inhibition of OATP1B1 and OATP1B3 with renal failure may impair the uptake and elimination of drugs that use these transporters, such as 7-Ethyl-10-hydroxycamptothecin (SN-38), used in cancer treatment [145,146].

### 4.4. Inorganic Phosphate Transporters (PiTs)

Type-III sodium-dependent phosphate transporters 1 and 2 (PiT-1 and PiT-2) are encoded by the *SLC20A1* and *SLC20A2* genes, respectively, part of the SLC20 gene family. PiTs structurally contain 12 transmembrane domains, with both NH_2_ and COOH terminations in the extracellular space, with transport based on the Na^+^ concentration gradient to translocate Pi [147,148]. Both transporters are related to Pi cellular uptake, with broad tissue distribution [148,149]. Studies suggest that PiTs are important for homeostasis but may also participate in pathological processes in response to hyperphosphatemia conditions [150,151].

Patients with CKD develop hyperphosphatemia, especially in the more advanced stages, which is related to several comorbidities in these patients [152]. Hyperphosphatemia conditions impair vascular functions, a process in which PiTs may play an important role [151,153,154]. Abbasian et al. (2015) demonstrated an increase in intracellular Pi concentrations in endothelial cells when the cells were exposed to hyperphosphatemia conditions, which was related to uptake by PiTs [154]. The study also showed that this effect was reversed with the inhibition and knockout of PiT-1 [154]. High Pi levels induced osteochondrogenic differentiation in VSMCs and matrix mineralization via PiT-1, which contributes to vascular calcification [151,155,156].

Another important Pi transporter is NaPi2B which is encoded by the *SLC34A2* gene, part of the SLC34 family. This transporter is responsible for the uptake of Pi in the intestine [148]. Nicotinic acid and nicotinamide are compounds that inhibit NaPi2B expression, reducing the intestinal absorption of Pi [157]. However, clinical and in vivo studies with these compounds have indicated a modest reduction in serum Pi levels and several adverse effects [152].

A synthesis of in vitro, animal, and clinical studies that link uremic toxins with cellular influx transporters, especially in CKD, is presented in Table 2.

### 4.5. Multidrug and Toxin Extrusion (MATE)

Multidrug and toxin extrusion (MATEs) are membrane proteins which are part of the SLC47 family, which is included in the SLC superfamily. The main representatives are MATE1 and MATE2-K, encoded by the *SLC47A1* and *SLC47A2* genes, respectively. Both mediate the cellular efflux of various compounds, particularly organic cations, presenting a large overlap with OCT substrates [158,159]. Basically, the transport mechanism of MATEs is antiport with the exchange of protons and organic cations [159]. MATE1 and MATE2-K are mainly expressed in the kidneys, but MATE1 can also be found in the liver, adrenal gland, heart, and others [82,160]. MATE1 and MATE2-K are found in the apical membrane of renal cells in the kidneys and are important for tubular secretion [82].

MATEs are related to the mediated transport of both uremic toxins and various drugs used in clinical treatments. Studies have shown that both MATE1 and MATE2-K have creatinine and guanidines as substrates [159,161]. Gessner et al. (2018) investigated TMAO cell efflux via MATE1 using polarized monolayers of canine kidney cells (MDCK cells) [125]. The authors reported greater translocation of TMAO from the basal to the apical region in cells expressing MATE1 or MATE1 in conjunction with OCT2 [125]. It was also observed that trimethoprim, an antibiotic inhibitor of OCTs and MATEs, suppressed TMAO translocation in cells expressing both OCT and MATE1 [125]. Other drugs are also known to interact with MATE1, such as trospium and ondansetron [162,163]. Cimetidin and metformin are other examples of drugs that are substrates for both MATE1 and MATE2-K [159]. However, the use of drugs that compete for MATEs and their impact on uremic toxin levels is still unclear. Furthermore, the expression of MATEs may be altered under pathological conditions. Nephrectomized rats showed a reduction in Mate1 protein levels in both males and females [164].

### 4.6. Breast Cancer Resistance Protein (BCRP)

Breast cancer resistance protein (BCRP) is encoded by the *ABCG2* gene and forms homodimers in the cell membrane to efflux a wide variety of substrates, including uremic toxins [165]. BCRP belongs to the ABCG family, part of the ABC superfamily. This protein has 655 amino acids organized into six α-helices and a nucleotide-binding domain located at the NH_2_ termination in the cytoplasm [166,167]. Like other members of the ABC superfamily, its activity is dependent on ATP hydrolysis [166,167]. BCRP is found expressed in the kidney, intestine, liver, placenta, and blood vessels [168,169,170,171]. However, studies have reported a decrease in Bcrp expression in the kidneys of nephrectomized animals compared to the sham group, demonstrating that CKD may alter the expression of this transporter [172,173].

BCRP is widely known for its drug transport, being primarily related to the pharmacokinetics of drugs used in cancer therapies. In recent years, BCRP has been shown to interact with several uremic toxins, especially in the transport of these compounds from the intracellular compartment to the renal lumen in the tubular secretion process. Mutsaers et al. (2015) demonstrated that BCRP inhibition with KO143 leads to increased intracellular PCS levels, indicating that this uremic toxin is a substrate for the transporter [174]. In another study, Takada et al. (2018) reported that *Abcg2*-knockout mice with chronic kidney dysfunction had high plasma levels as well as low renal clearance of IS compared to the control group, suggesting that BCRP is essential for renal elimination of this uremic toxin [175]. It was recently shown that BCRP inhibition with the uricosuric agent febuxostat resulted in decreased renal clearance of the IS [97]. Other studies using an in vitro model also suggest that IS, kynurenic acid, TMAO, and uric acid are BCRP substrates [110,175,176,177,178,179]. In addition, IS increased the expression of BCRP in intestinal cells, also resulting in greater translocation of urate from the basolateral to the apical compartments [179]. In fact, BCRP is an important uric acid transporter, contributing to the excretion of this uremic toxin by both the renal and intestinal routes. The increased plasma uric acid levels in patients with hyperuricemia may be correlated with polymorphisms in the *ABCG2* gene [180,181,182]. Other cells that express BCPR are also affected by uric acid. Komori et al. (2018) reported that uric acid reduced the protein levels of BCRP in the membrane of endothelial cells, resulting in lower efflux and increased intracellular concentrations of this uremic toxin [183].

### 4.7. Multidrug Resistance-Associated Proteins (MRPs)

Multidrug resistance-associated proteins (MRPs) are cell efflux transporters that interact with a wide variety of substrates. MRPs are part of the ABCC gene family which belongs to the ABC superfamily, characterized by ATP hydrolysis-dependent activity. The main members of this group are MRP2 and MRP4 encoded by the *ABCC2* and *ABCC4* genes, respectively. Both transporters are found in the apical membrane of proximal tubule cells, participating in the tubular secretion of endogenous and exogenous compounds. Moreover, MRP2 and MRP4 are also found in the liver, intestine, and brain capillary endothelium [168,184,185,186,187]. MRP2 expression increased in the kidneys and liver in a CKD animal model, while it decreased in the intestine [84,172,188]. On the other hand, MRP4 expression in nephrectomized animals did not change in the liver and intestine, while it is controversial in the kidneys, with studies indicating that it increases or does not change [84,172,189].

Although MRPs are considered potential transporters for uremic toxins, this interaction has been little explored. Mutsaers et al. (2011) observed that MRP4 activity is inhibited in the presence of IS, hippuric acid, kynurenic acid, IAA, and phenylacetic acid, which may suggest an interaction between these uremic toxins and the transporter [178]. In another study, Mutsaers et al. (2015) demonstrated that MRP4 activity was inhibited by PCS and *p*-cresyl glucuronide; however, the uptake of these toxins in vesicles expressing MRP4 was not observed [174]. In addition, Teft et al. (2017) showed that MRP2 and MRP4 participate in TMAO cell efflux in an in vitro model [110]. Like other transporters, MRP2 and MRP4 also have drugs as substrates, such as methotrexate [190]. Furthermore, studies have shown that several nonsteroidal anti-inflammatory drugs inhibited MRPs activity [190,191,192]. Therefore, more studies are needed to understand how the cellular efflux of uremic toxins occurs and its potential relationship with the use of prescribed drugs.

The main findings from in vitro and animal studies that investigated uremic toxins and their relationship to cellular efflux transporters are listed in Table 3.

## 5. Final Considerations

Uremic toxins are important factors contributing to the pathogenesis of CKD, and their elimination by dialysis therapies is limited. PBUTs specifically have a high affinity for serum albumin, and their removal by dialysis is still a challenge, although some studies have explored strategies that break this interaction, including using drugs that also have an affinity for albumin. Uremic toxins reach various tissues and organs through the bloodstream. Several uremic toxins are capable of interacting with membrane transporters, mediating their entry or exit from the cell and may cause biological effects under uremic conditions, such as cell dysfunction. Membrane transporters are particularly important in renal clearance and elimination by other routes of uremic toxins and drugs. Therefore, there may be competition between toxins and drugs as substrates of the transporter or even inhibition of its activity. Another relevant outcome is the change in the expression of these transporters in CKD. Consequently, tissue disposition and the elimination of uremic toxins as well as drugs may be affected. Despite this, clinical studies addressing this issue are still scarce. Altogether, it is important to evaluate the interaction of drugs and uremic toxins via albumin or transporters and their impact on the clinical course of patients with CKD. In addition, studies in this area could contribute to the development of new therapeutic strategies to improve the removal of uremic toxins by dialysis.

## Figures and Tables

**Table 1:** Cell membrane transporters that interact with uremic toxins and drugs.

Protein	Gene	Tissue Distribution	Uremic Toxin Interaction	Drug Interaction
OAT1	*SLC22A6*	Kidney	PCS, IS, kynurenic acid, hippuric acid	Probenecid, β-lactam antibiotics, nonsteroidal anti-inflammatory drugs
OAT2	*SLC22A7*	Kidney, liver	Creatinine	
OAT3	*SLC22A8*	Kidney	PCS, IS, kynurenic acid, hippuric acid	Probenecid, ciprofloxacin, β-lactam antibiotics, nonsteroidal anti-inflammatory drugs
OCT2	*SLC22A2*	Kidney	Creatinine, TMAO, methylguanidine, guanidine, putrescine	Metformin, cisplatin, cimetidine, vandetanib, trimethoprim
OCT3	*SLC22A3*	Choroid plexus, skeletal muscle, placenta, kidney	Creatinine	
OATP4C1	*SLCO4C1*	Kidney	ADMA	OATP4C1 expression is modulated by statins
PiT-1	*SLC20A1*	Endothelial cells, intestine, bones	Pi	
PiT-2	*SLC20A2*	Endothelial cells, intestine, bones, kidney	Pi	
NaPi2B	*SLC34A2*	Intestine	Pi	Nicotinic acid and nicotinamide inhibit NaPi2B expression
MATE1	*SLC47A1*	Kidney, liver, heart	TMAO, creatinine, guanidine	Trimethoprim, trospium, ondansetron
MATE2-K	*SLC47A2*	Kidney	Creatinine, guanidine	
BCRP	*ABCG2*	Kidney, intestine, blood vessels, placenta	PCS, IS, kynurenic acid, TMAO, uric acid	Febuxostat
MRP2	*ABCC2*	Kidney, liver, intestine, brain capillary endothelium	TMAO	Methotrexate, nonsteroidal anti-inflammatory drugs
MRP4	*ABCC4*	Kidney, liver, intestine, brain capillary endothelium	TMAO	Methotrexate, nonsteroidal anti-inflammatory drugs

Abbreviations: ADMA, asymmetric dimethylarginine; IS, indoxyl sulfate; PCS, *p*-cresyl sulfate; Pi, inorganic phosphate; TMAO, trimethylamine-*N*-oxide.

**Table 2:** Studies that address the relationship between cellular influx transporters and uremic toxins in chronic kidney disease.

Protein	Experimental Model	Main Findings
OAT1	OAT1-expressing HEK293 cells	Cell uptake of PCS [100]
	*Slc22a6*-knockout mice	Increased plasma levels of PCS, IS, and kynurenine [89]
	*Slc22a6*-knockout mice	Increased plasma levels of PCS, IS, and IAA [88]
	Rats	Renal uptake of hippurate, IAA, and IS [92]
	Nephrectomized rats	Decreased protein levels in the kidneys [83]
	Nephrectomized rats	Decreased protein and mRNA levels in the kidneys [84]
	Nephrectomized rats	Decreased protein levels in the kidneys [86]
	Nephrectomized rats	No differences in protein levels in the kidney [85]
	Nephrectomized rats treated with IS	Increased protein levels in the renal tubules [87]
OAT3	OAT3-expressing HEK293 cells	Cell uptake of PCS [100]
	Rats and Oat3-expressing oocytes	Renal uptake of IS [94]
	*Slc22a8*-knockout mice	Increased plasma levels of PCS, IS, CMPF, and TMAO [89]
	Rats	Renal uptake of IS and CMPF [92]
	Rats treated with IS	Decreased the renal clearance of IS through inhibition on the OAT3-mediated transport with ciprofloxacin [107]
	Nephrectomized rats	Decreased protein levels in the kidneys [86]
	Nephrectomized rats	No differences in protein levels in the kidney [85]
OAT1/3	HK-2 cells and rat renal cortical slices	Cell uptake of PCS, which was inhibited with probenecid, an inhibitor of OATs [93]
	Endothelial cells	Cell uptake of PCS and IS, which was inhibited with probenecid [101]
	Endothelial cells	Probenecid attenuated the inductive effects of IS on the expression of E-selectin and monocytic cell adhesion [103]
	Endothelial cells and aortic smooth muscle cells	Probenecid reversed the inductive effect of PCS on MCP-1 expression in endothelial cells and on the expression of osteogenic differentiation genes in aortic smooth muscle cells [102]
	Osteoblasts	Probenecid restored IS-induced effects on cell viability and ROS levels [104]
	Myoblast cells	Probenecid reversed IS-induced effects on ROS levels and inflammatory cytokine expression [106]
	Human subjects	Subjects treated with probenecid had elevated IS and kynurenine levels [111]
	Kidney transplant patients	Increased plasma levels of IS, PCS and IAA in patients with a prescription of at least one drug which inhibits OAT1/OAT3 [112]
OAT2	MDCKII cells	Cell uptake of creatinine [113]
	OAT2-transfected HEK cells	Cell uptake of creatinine [114]
OCT2	ciPTEC cells	Uptake of cationic uremic toxins, such as guanidine, methylguanidine, and putrescine [116]
	HEK293 cells	Cell uptake of guanidine compounds [118]
	HEK293 cells	Cell uptake of creatinine [120]
	MDCKII and HEK cells	Cell uptake of TMAO and transcellular transport [125]
	HEK293 cells	Cell uptake of putrescine [124]
	OCT2-expressing HEK cells	Inhibited by creatinine, dimethylamine, malondialdehyde, trimethylamine, homocysteine, indoxyl-β-d-glucuronide, and glutathione disulfide [123]
	HEK293 cells	Vandetanib inhibited the uptake of creatinine [129]
	*Slc22a2/1*-double knockout mice and HeLa cells	Increased plasma levels of TMAO. In vitro, TMAO transport [110]
	*Slc22a2/1*-double knockout mice and Oct2-transfected HEK293 cells	Increased plasma levels of TMAO. In vitro and In vitro, TMAO uptake [126]
	Nephrectomized rats	Decreased protein levels in the kidney [85]
	Patients with CKD and nephrectomized rats	Decreased protein levels in the kidney [130]
	Patients with cancer undergoing treatment with cisplatin and HEK293 cells	Increased serum levels of creatinine. In vitro, creatinine uptake [128]
	Patients with end-stage renal disease	Relationship between *SLC22A2* polymorphisms and phenotypes of net tubular creatinine secretion [119]
OATP4C1	MDCK cells	Transport of ADMA [141]
	HEK293 cells	Cell uptake of ADMA [140]
	HK-2 cells and rats treated with IS	IS reduced the OATP4C1 expression [143]
	Transgenic mice overexpressing OATP4C1 in the kidneys	Decreased plasma levels of ADMA, guanidino succinate, and *trans*-aconitate [142]
	Nephrectomized rats	Decreased mRNA levels in the kidney [144]
OATP1B1/3	Human hepatocytes and HEK293 cells	Decreased mRNA levels in cells exposed to uremic plasma. Inhibited by uremic toxin mix (IS, indole acetate, hippuric acid, and CMPF) [145]
PiT-1/2	Endothelial cells	Inhibition and knockout of PiT-1 reduced intracellular Pi concentrations [154]
	PiT-1-expressing oocytes	Pi transport [150]
	VSMCs	Uptake of Pi, which at high levels induces osteochondrogenic differentiation of VSMCs [155]
	Human smooth muscle cells	Cell uptake of Pi [156]

Abbreviations: ADMA, asymmetric dimethylarginine; ciPTEC, immortalized proximal tubule epithelial cells; CMPF, 3-Carboxy-4-methyl-5-propyl-2-furanpropionate; HEK, human embryonic kidney cells; HK-2, human proximal tubular cells; IAA, indole-3-acetic acid; IS, indoxyl sulfate; MCP-1, monocyte chemoattractant protein-1; MDCKII, Madin–Darby canine kidney II cells; PCS, *p*-cresyl sulfate; Pi, inorganic phosphate; ROS, reactive oxygen species; TMAO, trimethylamine-*N*-oxide; VSMCs, vascular smooth muscle cells.

**Table 3:** Studies that address the relationship between cellular efflux transporters and uremic toxins in chronic kidney disease.

Protein	Experimental Model	Main Findings
MATE1	HEK293 cells	Creatinine and guanidine as substrates [159]
	HEK293 cells	Creatinine as substrate [161]
	MDCKII and HEK cells	Transport of TMAO, which was suppressed by trimethoprim [125]
	Nephrectomized rats	Decreased protein levels in the kidneys [164]
MATE2-K	HEK293 cells	Creatinine and guanidine as substrates [159]
BCRP	ciPTEC cells	BCRP inhibition increased intracellular PCS levels [174]
	HeLa cells	TMAO transport [110]
	Membrane vesicles from MRP4-overexpressing HEK cells	Inhibited by hippuric acid, IS, and kynurenic acid [178]
	Caco-2 cells	Urate transport. IS reduced BCRP expression [179]
	Endothelial cells	Uric acid decreased the BCRP protein levels [183]
	*Abcg2*-knockout mice with adenine-induced CKD and membrane vesicles from HEK293A cells	Increased plasma levels and decreased renal elimination of IS. In vitro, IS transport [175]
	*Abcg2*-knockout mice and HEK293 cells	Kynurenic acid as substrate [177]
	*Abcg2*-knockout mice	Increased plasma levels and low urine levels of IS [175]
	Adenine-induced acute renal failure rats	Febuxostat, an BCRP inhibitor, decreased renal clearance of the IS [97]
	Nephrectomized rats	Decreased mRNA levels in the kidney [172]
	Nephrectomized rats	Decreased protein and mRNA levels in the kidney [173]
MRP2	HeLa cells	Performs cellular efflux of TMAO [110]
	Nephrectomized rats	Increased protein and mRNA levels in the kidneys [84]
	Nephrectomized rats	Increased mRNA levels in the liver and the kidneys [172]
	Nephrectomized rats	Decreased protein levels in the intestine [188]
MRP4	Membrane vesicles from MRP4-overexpressing HEK cells	Inhibited by IS, hippuric acid, kynurenic acid, IAA, and phenylacetic acid [178]
	ciPTEC cells	Inhibited by PCS and *p*-cresyl glucuronide [174]
	HeLa cells	Performs cellular efflux of TMAO [110]
	Nephrectomized rats	Increased protein and mRNA levels in the kidneys [84]
	Nephrectomized rats	No differences in mRNA levels in the kidney, liver, and intestine [172]
	Nephrectomized rats	No differences in mRNA levels in the kidney and the liver [189]

Abbreviations: ciPTEC, immortalized renal proximal tubule epithelial cells; HEK, human embryonic kidney cells; HRPTEC, human renal proximal tubule epithelial cells; IAA, indole-3-acetic acid; IS, indoxyl sulfate; MDCKII, Madin–Darby canine kidney II cells; PCS, *p*-cresyl sulfate; TMAO, trimethylamine-*N*-oxide.
